# Small-Molecule Factor Xa Inhibitors: Translational SAR, Assay-Aware Data Quality, and QSAR-Readiness for CADD-Oriented Discovery

**DOI:** 10.3390/ph19071017

**Published:** 2026-06-30

**Authors:** Paweł Gordon, Michał Janiak, Katarzyna Mądra-Gackowska, Lidia Wydeheft, Iga Hołyńska-Iwan, Marcin Gackowski

**Affiliations:** 1 University of Health Sciences in Bydgoszcz, Jagiellońska 4 Str., 85-067 Bydgoszcz, Poland; p.gordon@wsnoz.edu.pl; 2 Department of Toxicology and Bromatology, Faculty of Pharmacy, Ludwik Rydygier Collegium Medicum in Bydgoszcz, Nicolaus Copernicus University in Torun, Jurasza 2 Str., 85-089 Bydgoszcz, Poland; michal.janiak@doktorant.umk.pl; 3 Doctoral School of Medical and Health Sciences, Ludwik Rydygier Collegium Medicum in Bydgoszcz, Nicolaus Copernicus University in Torun, Jagiellońska 13-15 Str., 85-067 Bydgoszcz, Poland; 4 Department of Geriatrics, Faculty of Health Sciences, Ludwik Rydygier Collegium Medicum in Bydgoszcz, Nicolaus Copernicus University in Torun, Skłodowskiej Curie 9 Str., 85-094 Bydgoszcz, Poland; katarzyna.madra@cm.umk.pl (K.M.-G.); lidia.wydeheft@gmail.com (L.W.); 5 Department of Pathobiochemistry and Clinical Chemistry, Faculty of Pharmacy, Ludwik Rydygier Collegium Medicum in Bydgoszcz, Nicolaus Copernicus University in Torun, Skłodowskiej Curie 9 Str., 85-094 Bydgoszcz, Poland; igaholynska@cm.umk.pl

**Keywords:** factor Xa, FXa inhibitors, anticoagulants, medicinal chemistry, QSAR-readiness, computer-aided drug design, structure–activity relationship, anthranilamides, natural products, isosteviol

## Abstract

Factor Xa (FXa) remains a clinically validated and chemically tractable anticoagulant target despite the therapeutic role of direct oral FXa inhibitors. Contemporary FXa inhibitor literature, however, is heterogeneous in scaffold design, endpoint reporting, assay consistency, translational depth, and suitability for computer-aided drug design (CADD). This review evaluates published series of small-molecule FXa inhibitors through a framework that combines translational structure–activity relationships (SARs), assay-aware data quality, and QSAR-readiness. A structured narrative synthesis focused mainly on post-2014 studies reporting discrete small-molecule or semisynthetic FXa inhibitors. Eligible series were classified as fully synthetic or natural-product-derived/semisynthetic chemotypes, and extraction covered scaffold architecture, potency endpoints, assay context, selectivity, clotting or antithrombotic readouts, PK/ADME, structural clarity, translational context, and extraction confidence. QSAR-readiness was assessed using analog density, congenericity, endpoint quality, assay comparability, activity range, structural interpretability, and curation burden. Fully synthetic chemotypes, particularly anthranilamide-derived and related scaffolds, provided the most coherent and modellable FXa datasets, whereas natural-product-derived and semisynthetic series expanded structural diversity. Many exploratory series, however, were limited by small analog sets, heterogeneous endpoints, incomplete translational characterization, narrow activity ranges, or higher curation burden. The practical value of published FXa inhibitor series, therefore, depends not only on potency but also on whether chemical and biological information can be reconstructed with confidence for reproducible SAR interpretation, local QSAR modeling, AI/ML-enabled CADD reuse, and clinical benchmark-aware prioritization. The QSAR-readiness framework is a critical triage tool, not a substitute for formal validation, distinguishing datasets suitable for curated local modeling from those better suited to qualitative SAR, scaffold inspiration, or translational hypotheses.

## 1. Introduction

Factor Xa (FXa) occupies a uniquely strategic position in the coagulation cascade because it sits at the junction of the intrinsic and extrinsic pathways and contributes to thrombin generation, fibrin formation, and clot stabilization [1,2,3]. This mechanistic role has made FXa a clinically validated target for anticoagulant therapy, as reflected in the development of direct oral FXa inhibitors such as rivaroxaban and apixaban [2,3,4]. The success of these agents has confirmed that selective small-molecule inhibition of FXa can deliver meaningful therapeutic benefit, but it has not closed the field. On the contrary, contemporary medicinal chemistry programs show that FXa remains chemically designable, particularly when optimization extends beyond enzymatic potency toward selectivity, clotting readouts, pharmacokinetic behavior, and translational pharmacology [5,6,7,8,9,10,11,12], while earlier structural programs remain important for understanding binding-site design principles [13].

The imperative for such multidimensional optimization is particularly pronounced in older adults, who represent a major real-world population for FXa-targeted anticoagulation. Atrial fibrillation is highly prevalent in aging populations and represents a major indication for anticoagulation [14,15], while the incidence of venous thromboembolism likewise rises markedly with advancing age [16,17]. However, older patients rarely constitute a straightforward pharmacological target population. Clinical factors such as frailty, multimorbidity, age-related renal impairment, polypharmacy, and bleeding vulnerability substantially increase treatment complexity [18,19,20,21]. Consequently, the development of next-generation FXa inhibitors must look beyond isolated enzymatic potency to systematically address selectivity against related serine proteases and predictable pharmacokinetic behavior, and minimize the risk of drug–drug interactions [3,18,19,20,22].

The medicinal chemistry literature over the last decade reflects this translational complexity. Structural and SAR-guided studies of FXa inhibitors have consistently highlighted the critical topography of the S1 and S4 pockets, emphasizing the role of neutral P1 motifs, aromatic-box recognition, and linker geometry in achieving high-affinity inhibition alongside oral bioavailability [2,13,23,24]. To this end, recent synthetic programs have explored anthranilamide derivatives, heterocyclic scaffolds, and structurally constrained small-molecule series, frequently validating their potent enzymatic activity with translational clotting or antithrombotic readouts [5,6,7,8,11,12]. In parallel, natural-product-derived and semisynthetic approaches, including isosteviol-based scaffolds and diterpenoid-derived compounds, have been investigated to provide alternative scaffold topologies and expand the accessible stereochemical and structural diversity for FXa recognition [9,10,25,26].

While this chemical diversification expands design opportunities, it also introduces important challenges for computer-aided drug design (CADD) and quantitative structure–activity relationship (QSAR) workflows. Fully synthetic inhibitor series typically yield congeneric and experimentally coherent datasets that can support systematic local modeling [5,6,7,8,11,12]. In contrast, natural-product-derived or semisynthetic series, despite their structural novelty, are frequently characterized by smaller, less standardized datasets [9,10,25,26]. Crucially, reported biological activities across the FXa literature range from precise enzymatic endpoints (IC_50_ or K_i_ values) to exploratory, screening-style percent-inhibition readouts obtained under heterogeneous assay conditions [9,10,12,26,27]. Because these academic studies differ fundamentally in assay formats, endpoint definitions, and levels of biological characterization, cross-study comparison is highly challenging. Cheminformatics research has long demonstrated that establishing QSAR-readiness and developing robust predictive models depend on consistent endpoints and rigorous chemical data curation [28,29,30]. Conversely, relying on uncurated or heterogeneous datasets compromises the reliability of docking, virtual screening, and structure-guided optimization [29,30,31].

Existing reviews of FXa inhibitors have primarily focused on descriptive scaffold classifications, the historical evolution of medicinal chemistry, or the clinical development of established direct oral anticoagulants [1,3,22,32]. While valuable for basic orientation, these potency- and scaffold-centered catalogs typically do not evaluate whether published datasets are sufficiently coherent, biologically interpretable, and experimentally consistent to serve as reliable inputs for computational workflows [28,29,30,31]. The present review addresses this gap by examining small-molecule FXa inhibitors reported over the last decade through three integrated lenses: translational SARs, assay-aware data quality, and QSAR-readiness. By distinguishing dataset qualities between fully synthetic and natural-product-derived chemotypes, this work establishes a practical framework for CADD-driven discovery and rational scaffold prioritization. Ultimately, this approach aims to better align early-stage medicinal chemistry strategies with real-world clinical constraints, particularly those dictated by anticoagulant safety profiles in older patients [14,17,18,19,20,21].

## 2. Scope and Inclusion Strategy

### 2.1. Search Scope and Eligibility Criteria

This review was conducted as a structured, assay-aware narrative synthesis rather than a formal systematic review or meta-analysis. Eligibility criteria and data-extraction fields were predefined to improve transparency and support consistent comparison of FXa inhibitor series across medicinal chemistry, translational, and QSAR-readiness domains. The literature scope was centered on the post-2014 period, because this timeframe reflects the contemporary phase of FXa inhibitor optimization, in which programs are increasingly evaluated not only by potency, but also by translational pharmacology, developability, and differentiation from established oral anticoagulants [32]. Peer-reviewed primary research articles constituted the main evidence base. In contrast, earlier seminal papers and selected landmark reviews were used to provide background on target biology, binding-site interactions, and the historical evolution of FXa-directed medicinal chemistry [2,3,4,22,23,24,32].

Literature was identified through targeted searches of PubMed/MEDLINE, Scopus, Web of Science, and Google Scholar using combinations of the terms “factor Xa” OR “FXa”, “inhibitor”, “small molecule”, “medicinal chemistry”, “structure–activity relationship” OR “SAR”, “QSAR”, “computer-aided drug design” OR “CADD”, “docking”, “anticoagulant”, “direct oral anticoagulant”, and “serine protease inhibitor”. The primary search window was January 2014 to May 2026; earlier landmark papers were retained only when needed for target biology, marketed-agent context, binding-site logic, or QSAR methodology. The final evidence base comprised representative post-2014 experimental reports on small-molecule or semisynthetic FXa inhibitors, supplemented by selected clinical, structural, and methodological sources. At the series level, the evidence map included 15 post-2014 experimental reports on small-molecule or semisynthetic FXa inhibitors and two retrospective or computational follow-up records used to contextualize QSAR/CADD reuse. Because this review was designed as a targeted narrative synthesis rather than a formal systematic review, we did not generate a PRISMA-style flow diagram or exhaustive record count; the evidence map should therefore be interpreted as a curated series-level selection.

Eligible studies were required to report discrete small molecules evaluated against FXa in enzymatic, biochemical, clotting, or integrated pharmacological settings. This review excluded biologics, antibodies, aptamers, peptides developed primarily as large-molecule anticoagulants, formulation-driven studies without medicinal-chemistry progression, and purely computational studies lacking an experimentally validated inhibitor series. Crude natural extracts were not considered FXa inhibitor programs unless a defined active constituent, semisynthetic derivative set, or structurally interpretable lead series was reported. For organizational purposes, included chemotypes were assigned to one of two domains: fully synthetic series and natural-product-derived or semisynthetic series. Borderline cases were classified according to dominant design logic rather than the synthetic route alone. In practical terms, borderline cases were assigned according to the scaffold logic that dominated analog expansion and assay presentation: semisynthetic series retaining a recognizable bioscaffold were grouped with the natural-product-derived domain, whereas synthetic expansions using only natural-product-inspired motifs, without a retained bioscaffold, were grouped with the fully synthetic domain.

### 2.2. Multi-Layer Data Extraction and Translational Depth

Within each included series, data extraction extended beyond headline potency values to evaluate the underlying structure of the experimental endpoints. The first extraction layer focused on assay awareness, recording whether activity was reported as K_i_, IC_50_, pK_i_, pIC_50_, percentage inhibition at a single concentration, or censored values (e.g., “>10 μM”); whether assays employed purified human FXa or alternative enzyme sources; whether substrate systems and assay conditions appeared internally consistent; and whether selectivity was assessed against thrombin or other related serine proteases. This assay-aware layer is important because variability in endpoint definition and assay design can influence both medicinal chemistry interpretation and the suitability of data for downstream computational modeling [28,29,30,31].

A second extraction layer addressed translational depth. Where available, each series was annotated for clotting readouts such as prothrombin time (PT) and activated partial thromboplastin time (aPTT), ex vivo or in vivo antithrombotic activity, bleeding-related observations, and early pharmacokinetic/ADME (PK/ADME) properties. Although not all FXa inhibitor programs report these parameters, datasets that extend beyond isolated enzyme assays provide a more informative context for assessing whether structure–activity relationships remain relevant under pharmacologically meaningful conditions. This translational resolution is particularly relevant when aligning medicinal chemistry with real-world clinical profiles in older patients, in whom atrial fibrillation, venous thromboembolism, frailty, renal impairment, and polypharmacy substantially shape anticoagulant safety and therapeutic performance [14,18,19,20,21].

### 2.3. Operationalizing QSAR-Readiness

For this review, QSAR-readiness is treated as an operational concept grounded in established principles of QSAR modeling and chemical-data curation [28,29,30,31]. A dataset was considered QSAR-ready when it combined five core criteria:

▪ A sufficiently populated and chemically congeneric analog set;
▪ Exact and predominantly uncensored potency endpoints;
▪ Internally comparable assay conditions;
▪ Clearly defined chemical structures and stereochemistry;
▪ Limited ambiguity during data curation and descriptor generation before model development.

In this review, QSAR-readiness denotes suitability for local model development after appropriate curation; it does not imply that any individual published dataset or model automatically satisfies full best-practice validation requirements, including external validation, applicability-domain assessment, or robustness testing [28,29,30,31]. In addition, QSAR-readiness should be interpreted relative to the activity range of a series: even a chemically clean and assay-consistent dataset may have limited modeling value if the potency range is too narrow to support informative regression. Series that are structurally interesting but fragmented across multiple assay formats, dominated by single-point inhibition data, composed of only a few loosely related analogs, or insufficiently informative in activity range were retained for qualitative SAR interpretation but not classified as high-confidence local regression datasets [28,29,30,31]. This distinction is methodological rather than hierarchical: lower QSAR-readiness does not imply lower scientific value, but it defines the type of inference that can reasonably be drawn from the available evidence.

The main evidence-extraction domains used throughout this review are summarized in Table 1, and the working criteria for distinguishing levels of QSAR-readiness are presented in Table 2.

Low or intermediate readiness may result from any major gating limitation, including insufficient activity range, incomplete endpoint harmonization, structural ambiguity, limited confidence in data extraction, or insufficient evidence of common binding logic. The categories used here should be viewed as a pragmatic triage framework rather than as a formally weighted prediction score. They are intended to identify which published series can reasonably support local modeling after curation, and which should remain sources of qualitative SAR, scaffold inspiration, or translational context [28,29,30,31].

Table 3 relates the present QSAR-readiness framework to established QSAR data-quality guidance and clarifies that it is a review-level triage framework rather than a substitute for formal model validation. The comparison draws on established QSAR curation and validation concepts [28,29,30,31] and on the operational framework proposed in this review.

From a practical CADD perspective, we treat QSAR-readiness as a filtering step rather than as a descriptive label. A dataset that looks chemically attractive in a review table may still be unsuitable for descriptor-based modeling if key activity values, assay settings, stereochemical assignments, or activity variance are insufficiently defined. The most useful role of the framework is to prevent premature pooling and to direct each series toward the level of inference it can actually support: local QSAR, qualitative SAR, or scaffold inspiration. Meeting these criteria is only the starting point: even a high-readiness series still requires expert curation, endpoint verification, and an assessment of applicability domain before QSAR or ML output can be treated as decision-supporting evidence.

## 3. FXa Binding Logic Revisited

### 3.1. Why FXa Remains Chemically Designable

Although the catalytic machinery of FXa is conserved across serine proteases, medicinally useful inhibition is not driven by catalytic residues alone. Modern direct FXa inhibitors commonly exploit a bifocal recognition pattern spanning the deep S1 pocket and the more lipophilic S4 region, while the intervening scaffold controls binding geometry, conformational preorganization, and the balance between affinity, selectivity, and developability [2,4,13,23,24]. This binding logic helps explain why structurally diverse FXa inhibitor chemotypes can converge on a recognizable pharmacophoric architecture, and why post-DOAC FXa discovery remains a matter of fine chemical design rather than simple potency maximization [2,4,13,23,24]. The same pocket-oriented design logic is also evident in later exploratory families such as tetrahydropyrazolopyridones and 2,3-dihydroquinazolin-4(1H)-ones, which contribute chemically meaningful SAR while remaining better suited to qualitative SAR interpretation or limited local modeling than to broad standalone QSAR modeling [11,12,28,29,30,31].

### 3.2. The S1 Pocket: Potency Anchor and Property Liability

The S1 pocket remains a primary determinant of specificity in many FXa inhibitor programs. The bottom of the pocket is defined by Asp189, which historically favored strongly basic P1 groups capable of high-affinity electrostatic interactions. Although these early solutions often produced excellent potency, they could also introduce physicochemical liabilities, particularly reduced permeability and less favorable oral exposure. Structural studies later demonstrated that neutral or weakly basic P1 motifs could preserve high affinity when steric fit and hydrogen-bonding geometry were appropriately optimized, marking an important transition from highly potent early-generation inhibitor scaffolds toward orally bioavailable and pharmacologically balanced compounds [2,4,13,23,24].

For translational medicinal chemistry, the P1 decision is therefore never purely local. A strongly interacting S1 binder may improve enzymatic potency yet impair the broader physicochemical profile. In contrast, a neutral P1 solution may partially relax direct electrostatic anchoring while providing a more favorable platform for oral bioavailability, permeability, and consistency of exposure [2,4,13,23,24]. This trade-off is especially relevant in anticoagulant discovery, where chronic dosing, predictable pharmacokinetics, and safety margins often become as important as maximizing local binding interactions within the pocket architecture [2,3,4].

### 3.3. The S4 Aromatic Box as a Selectivity and Affinity Lever

If S1 often determines whether a series can achieve high potency, S4 strongly influences how that potency is translated into affinity, selectivity, and whole-molecule properties. The S4 subsite is commonly described as an aromatic box formed mainly by Tyr99, Phe174, and Trp215, and it accommodates many lipophilic or heteroaromatic elements used in advanced FXa inhibitors [2,4,13,23,24]. Productive S4 occupation can substantially increase affinity and may also help shape selectivity relative to related coagulation proteases, but this optimization is rarely property-neutral. Excessive reliance on lipophilic S4 interactions may increase plasma protein binding and complicate solubility, metabolic stability, pharmacokinetic behavior, or broader developability [13].

Accordingly, successful FXa medicinal chemistry has often treated S4 not merely as a potency booster, but as a tunable design handle [2,4,13,24]. Substituent shape, aromatic surface, heteroatom placement, and ring constraint can influence how efficiently the ligand fills the S4 pocket and whether the resulting gain in affinity remains compatible with a developable physicochemical profile [2,12,24]. This helps explain why apparently similar FXa inhibitor series may diverge substantially in practical value once the analysis extends beyond nanomolar enzyme inhibition.

### 3.4. The Intervening Region: Geometry, Preorganization, and Hidden SAR

A useful FXa inhibitor does not simply place one fragment in S1 and another in S4. The central linker or rigidified core determines whether these fragments are presented with the correct spacing, vector orientation, and conformational cost. In several structure-guided programs, potency improvement was associated with scaffold preorganization, restricted torsional freedom, and ligand geometry that maintained productive interactions within the FXa active site [2,4,13,24]. As a result, some of the most informative SAR in FXa discovery lies not only in pocket-filling substituents, but also in apparently modest changes to the bridging framework that reshape the overall binding pose.

This aspect is also central to QSAR interpretation. Two series may nominally share S1 and S4 motifs, yet if their cores impose different three-dimensional alignments, pooling them into a shared quantitative modeling framework may yield a structurally incoherent dataset. In other words, scaffold commonality alone is not sufficient; the underlying binding solution should also remain coherent at the three-dimensional level. This is one of the main reasons why the present review distinguishes between descriptive SAR continuity and genuinely modellable chemical space [28,29,30,31].

### 3.5. From Pocket Occupancy to Translational SAR

The practical lesson is that FXa binding logic cannot be reduced to a static representation of S1 and S4 occupancy. Medicinal chemistry decisions made at these sites propagate outward into selectivity, clotting readouts, antithrombotic efficacy, pharmacokinetics, and ultimately translational applicability. For programs intended for chronic oral anticoagulation, especially in older adults with atrial fibrillation, venous thromboembolism, multimorbidity, renal impairment, or polypharmacy, design quality depends on whether high-affinity binding remains compatible with predictable exposure, manageable pharmacological risk, and an interpretable safety–efficacy window under clinically relevant constraints [18,19,20,21]. Related translational work on engineered FXa variants that retain procoagulant activity in the presence of direct FXa inhibitors further illustrates that FXa-directed drug design is clinically linked not only to inhibition potency, but also to reversibility and bleeding-management considerations [38].

**Figure 1:**
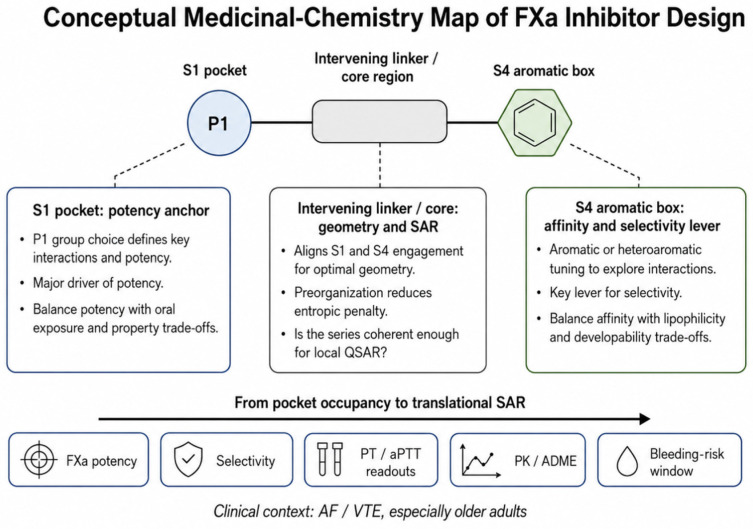
Conceptual medicinal chemistry map of FXa inhibitor design. The schematic links S1-pocket recognition, S4 aromatic-box engagement, and intervening linker/core geometry with downstream translational SAR features, including FXa potency, selectivity, PT/aPTT readouts, PK/ADME properties, and bleeding-risk considerations. It represents a synthesis based on structural and medicinal-chemistry studies of representative FXa inhibitors [2,4,5,6,7,8,9,10,11,12,13,23,24,25,26].

For that reason, the next sections do not classify chemotypes only by scaffold lineage. Instead, they examine how different solutions to the S1–S4 design problem translate into broader medicinal chemistry performance, biological depth, and downstream QSAR readiness. In this review, binding logic is therefore not treated as an isolated structural concept, but as a conceptual bridge linking pocket-level recognition to translational anticoagulant design. This logic is summarized schematically in Figure 1.

## 4. Fully Synthetic Chemotypes

Fully synthetic chemotypes remain a major reservoir of designable FXa chemical space. Compared with natural-product-derived series, synthetic programs more often provide larger analog matrices, clearer medicinal-chemistry intent, and a better chance of maintaining consistent assay formats across a series [5,6,7,8]. Although this does not necessarily guarantee translational depth, it improves the likelihood that the observed SAR reflects coherent pocket engineering rather than assay heterogeneity.

### 4.1. From Scaffold Novelty to Modellable Analog Series

Many post-2014 synthetic programs did not attempt to reinvent the xaban pharmacophore completely; rather, they rebalanced established design elements around alternative cores, P1 motifs, and linker solutions [2,4,32]. From a review perspective, this is advantageous: even when scaffold innovation remains incremental, the resulting datasets are often better suited to local QSAR-oriented analysis than fragmented collections of structurally distant hits. Several examples from the contemporary period of medicinal chemistry illustrate this point. Yang et al. reported isoxazolo[5,4-d]pyrimidin-4(5H)-one derivatives as antithrombotic agents and identified compound **6g** as a potent FXa inhibitor, with IC_50_ = 0.013 μM, 2 × PT = 2.12 μM in human plasma, and high selectivity over thrombin and trypsin [5]. In a parallel program, the same group described 3,4-diaminobenzoyl-based inhibitors and identified compound **7b** as a potent and selective direct FXa inhibitor with in vivo antithrombotic activity [6]. Sun et al. later described tetrahydropyrazolopyridone derivatives as FXa inhibitors; among them, compound **15c** showed an IC_50_(FXa) value of 0.14 μM, and selected derivatives were further evaluated in a rat venous thrombosis model [11]. These studies are methodologically valuable because they move beyond potency-only or purely in silico descriptions and connect chemotype modification with biological progression, including clotting or in vivo antithrombotic readouts alongside enzyme inhibition. Although the analog counts in these studies generally fall into the intermediate-readiness category according to the criteria used in this review, their combination of enzyme-inhibition data, selected clotting or in vivo readouts, and coherent scaffold modification provides a sufficient basis for meaningful SAR interpretation [5,6,11].

### 4.2. Anthranilamide Lineages as a Particularly Instructive Synthetic Platform

Among fully synthetic FXa chemotypes, anthranilamide-centered and anthranilamide-derived series are especially informative because they remain close to the established P1/core/P4 binding logic of direct FXa inhibitors while still tolerating substantial medicinal chemistry diversification [7,8,12,33,39]. Xing et al. used a fragment-based drug design (FBDD) strategy to identify anthranilamide derivatives as potential FXa inhibitors, demonstrating that compound **9b** displayed an FXa IC_50_ of 23 nM, excellent selectivity versus thrombin, and a 2 × PT value of 8.7 μM [7]. In subsequent structural optimization of this lineage targeting peripheral P1 and P4 configurations, compound **16g** emerged as a highly potent analog with a pronounced in vitro anticoagulant profile and documented cellular safety in H9C2 cells [8]. Wang et al. also described anthranilamide-based FXa inhibitors; compounds **1a**, **1g**, and **1s** showed nanomolar FXa inhibition with high selectivity over thrombin, whereas compounds **1g** and **1s** additionally prolonged PT in rat and human plasma [33]. The structural versatility of this platform is further exemplified by scaffold-hopping into closed heterocyclic variants, such as the 2,3-dihydroquinazolin-4(1H)-one cores reported by Xing et al., where candidates such as **8c** and **8e** maintained high selectivity over thrombin alongside plasma clotting readouts, including 2 × PT values [12].

From the perspective of the present review, the broader anthranilamide family is important for several reasons. First, it typically offers a recognizable common core with systematic peripheral variation, which is a prerequisite for local QSAR-oriented analysis. Second, these series are grounded in medicinal chemistry logic, directly linked to FXa pocket architecture, rather than in broad phenotypic screening. Third, representative studies in this lineage report exact inhibitory values along with selectivity, clotting readouts, in vivo antithrombotic data, bleeding-risk evaluation, or PK data, thereby improving biological interpretability and strengthening translational context [7,8,12,33]. Finally, the family illustrates how moderately sized or scaffold-modified branches can still provide meaningful design insight within the QSAR-readiness criteria used in this review, without necessarily being pooled into a single global model.

### 4.3. Piperazinylanthranilamides and the Value of Congeneric Expansion

A more recent extension of the anthranilamide theme is the piperazinylanthranilamide series reported by Khadse et al. in 2022 [35]. The accessible publication metadata describe a designed and synthesized series of piperazinylanthranilamide derivatives as potential FXa inhibitors, with substituted phenylpiperazinylamides introduced as S4-binding ligands to improve physicochemical properties. Compounds **6y** and **7f** were reported as the most active examples, with FXa IC_50_ values of 0.6 µM and 0.74 µM, respectively, high selectivity over thrombin, and good anticoagulant activity in rats [35]. The piperazinylanthranilamide series is chemically relevant and appears consistent with the broader anthranilamide design logic. However, in the present review, it was evaluated only at the level supported by accessible publication metadata, rather than through full series-level extraction. Its placement within the QSAR-readiness framework should therefore be regarded as provisional and primarily informative for qualitative SAR rather than definitive local QSAR classification [28,29,30,31,35]. This type of contribution remains valuable because it illustrates how anthranilamide-centered expansion can generate structurally interpretable FXa inhibitor series, even when the available information is insufficient for high-confidence quantitative reuse [5,6,7,8,35].

### 4.4. Potent but Smaller Exploratory Series: Useful SAR, Limited Modeling Depth

Not every fully synthetic study produces a dataset equally suitable for quantitative modeling. Rayani et al. reported a compact series of pyrazolyl piperidine analogs in 2022; compound **4a**, bearing a 4-chlorophenyl substituent, displayed the highest in vitro FXa inhibitory activity in that series, with IC_50_ = 13.4 nM, and PT/aPTT assays indicated anticoagulant activity in the reported system [37]. This study is a useful example of a pharmacologically interesting synthetic series that remains methodologically limited for broader QSAR-oriented modeling: the set comprises only eight analogs, which is insufficient to support a robust local regression model without a substantial risk of overfitting [28,29,30,31,37]. A conceptually different but equally instructive case is provided by the 2024 dual FXa/XIa hybrid series of Skoptsova et al., who synthesized 5,6-dihydro-4H-pyrrolo[3,2,1-ij]quinolin-2(1H)-one derivatives and evaluated them against both coagulation factors, including thrombin counter-screening for selected compounds [27]. From a drug-design perspective, this work probes whether hybrid architectures can broaden anticoagulant target coverage while retaining FXa activity. From a QSAR perspective, however, the dual-target optimization goal and exploratory dataset structure make the series less suitable for a clean local FXa model than for qualitative SAR interpretation. Such studies remain valuable as hypothesis-generating case material rather than as directly modellable FXa datasets [27,28,29,30,31]. Even apparently modellable series require caution: without sufficient analog coverage and validation beyond internal statistics, good-looking QSAR results may still reflect overfitting rather than transferable structure–activity information.

### 4.5. What Synthetic Chemotypes Contribute to Translational SAR

Taken together, the fully synthetic literature shows that FXa medicinal chemistry after DOAC validation has not been a simple exercise in increasing potency. Instead, contemporary synthetic programs illustrate how pocket-level design, assay consistency, and at least partial translational readouts can be integrated within coherent medicinal-chemistry frameworks [2,3,4,5,6,7,8,11,12,33]. Within this domain, three broad patterns recur. First, anthranilamide-centered, anthranilamide-derived, and cyclized anthranilamide-related scaffold families provide a consistent bridge between structural reasoning and series-level optimization [7,8,12,33,39]. Second, many reviewed synthetic programs report exact potency values and internally comparable assay formats, which improves their suitability for local SAR interpretation and QSAR-readiness assessment [5,6,7,8,11,12,28,29,30,31,33]. Third, the most informative well-characterized studies extend beyond isolated enzyme inhibition and report selectivity, clotting assays, in vivo antithrombotic data, bleeding-risk evaluation, or pharmacokinetic information, enabling interpretation in a translational rather than purely enzyme-centered context [5,6,7,8,11,12,33]. From a practical standpoint, fully synthetic chemotypes therefore constitute the principal source of high- and intermediate-readiness FXa datasets. They provide a methodological reference point for assessing the extent to which natural-product-derived and semisynthetic series can support translational SAR interpretation and QSAR-oriented analysis. Representative examples from this fully synthetic domain are summarized in Table 4. To improve practical comparison between scaffold classes, the table emphasizes representative compounds and key quantitative activity or clotting values when reported in the primary literature. These values should be read as scaffold-level benchmarks rather than as directly cross-assay potency rankings. To preserve this comparative focus, the table uses representative compound identifiers as structure anchors, allowing readers to trace the corresponding molecular drawings in the cited primary reports without overloading the table with multiple 2D structures.

The fully synthetic evidence map is not restricted to anthranilamide-centered scaffolds. It also includes isoxazolo[5,4-d]pyrimidin-4(5H)-ones, 3,4-diaminobenzoyl derivatives, tetrahydropyrazolopyridones, compact pyrazolyl piperidines, and dual FXa/XIa pyrroloquinolinone hybrids [5,6,11,27,37]. These examples illustrate that chemically relevant and pharmacologically promising FXa series can differ substantially in QSAR-readiness due to analog density, endpoint coverage, assay structure, and translational follow-up. This distinction matters because synthetic origin and even nanomolar potency should not be treated as automatic evidence of computational usability. Small but potent sets, dual-target exploratory programs, or series with incomplete endpoint reporting may be excellent medicinal-chemistry starting points but weak training sets for QSAR or ML. In such cases, local qualitative SAR is more defensible than forcing a formal predictive model.

## 5. Natural-Product-Derived and Semisynthetic Chemotypes

Natural-product-derived and semisynthetic FXa chemotypes occupy a smaller and more heterogeneous evidence space than fully synthetic series. Their value lies less in generating large, uniformly optimized analog matrices and more in introducing bioscaffold-driven topology, stereochemical richness, and alternative vectors for tuning ligand–protein interactions [9,10,25,26]. In practice, the strongest examples, particularly isosteviol-derived series, can provide exact FXa potency values, selectivity data, docking or SPR support, clotting readouts, pharmacokinetic information in selected cases, and ex vivo or in vivo antithrombotic evidence [9,10]. However, not all natural-product-derived reports reach this level of biological or assay depth, and some remain better suited for qualitative SAR interpretation than for high-confidence local QSAR modeling [25,26]. This evidence asymmetry does not diminish the conceptual importance of these chemotypes. It does, however, imply that they should be interpreted with different evidentiary expectations than the more congeneric synthetic series discussed previously.

### 5.1. Isosteviol as the Most Instructive Semisynthetic FXa Platform

Within the last decade, isosteviol-based chemistry has emerged as one of the most instructive semisynthetic bridges between natural-product inspiration and FXa-oriented medicinal chemistry. Chen et al. reported a potent isosteviol-derived antithrombotic lead, compound **6k**, that selectively inhibited FXa with K_i_ = 0.015 μm, prolonged ex vivo PT and aPTT, inhibited ADP-induced platelet aggregation in rats, and showed ex vivo and in vivo antithrombotic activity [9]. In a related follow-up study, Shi et al. described a focused series of isosteviol derivatives; compounds **22**, **35**, and **38** showed stronger FXa inhibitory activity than isosteviol. Selected compounds were evaluated by surface plasmon resonance, and compounds **22** and **35** displayed moderate-to-high anticoagulant activity with comparable effects on PT and aPTT [10]. Taken together, these studies suggest that isosteviol can function as more than a bioactive starting point; it represents a semisynthetic platform capable of supporting structure-informed SAR optimization [9,10].

### 5.2. Beyond Isosteviol: Diversity and Modeling Constraints in Diterpenoids

Isosteviol is not the only diterpenoid-derived lineage of interest. Wang et al. reported a semisynthetic series of ent-norstrobane diterpenoids, demonstrating that scaffold rearrangement within a natural diterpene framework can generate compounds with measurable FXa inhibition; compound **7** showed the strongest FXa inhibitory activity in that series, with IC_50_ = 81 ± 11 nM [25]. In a related pimarane-derived study, the same group prepared epoxy-pimarane derivatives and observed moderate FXa inhibitory activity, with reported IC_50_ values ranging from 0.22 to 27.9 μm, using edoxaban as a positive control [26]. These studies extend the natural-product-derived design space, but they also illustrate why non-isosteviol semisynthetic diterpenoid reports are generally better suited for qualitative SAR and scaffold-level hypothesis generation than for high-confidence local QSAR modeling: analog sets are small, translational readouts are sparse, and the resulting SAR remains insufficiently populated for robust local modeling [25,26].

### 5.3. Coumarin-Based Systems: Chemically Familiar, Mechanistically More Fragmented

Coumarin-based systems present a distinct, more cautious scenario. Recent work on newly synthesized 4,7-dihydroxycoumarin derivatives examined their potential to inhibit coagulation factors XIIa, Xa, and IIa, thereby broadening the mechanistic context of coumarin-derived coagulation factor inhibition [36]. At the same time, the available report appears to describe a very limited set of derivatives with a multi-target coagulation profile, which makes this study useful for hypothesis generation and structural exploration, but not for local QSAR in the strict sense as applied in this review [28,29,30,31,36]. In other words, studies on coumarin-derived FXa are scientifically informative. Yet they currently contribute more to scaffold-level insight than to modellable analog-series design—a pattern methodologically similar to other small, exploratory, natural-product-derived or natural-product-related reports [25,26,36].

### 5.4. What Natural-Product-Derived Chemotypes Contribute to Translational SAR

The natural-product-derived literature complements the fully synthetic domain by contributing different types of design information. It less often supplies large or highly standardized datasets. Still, it can reveal alternative design opportunities that are less apparent in highly optimized xaban-like frameworks, particularly regarding three-dimensionality, scaffold rigidification, stereochemical complexity, and noncanonical substituent vectors [9,10,25,26]. For translational SAR, this distinction is important because novelty can be valuable even when it does not improve potency; it may also reshape physicochemical properties, target selectivity, clotting activity, or broader developability [9,10]. For consistency with Table 4, Table 5 uses representative compound identifiers as structure anchors, allowing readers to trace the corresponding molecular drawings in the cited primary reports while keeping the table focused on biological endpoints and QSAR-reuse implications. Such unique structures often remain underexplored rather than intrinsically unsuitable for QSAR/CADD. Their present limitations are usually the small number of analogs, incomplete activity annotation, or incomplete translational characterization, rather than the bioscaffold concept itself.

The more cautious treatment of these series should therefore be read as criticism of the dataset structure rather than of natural-product chemistry. In practice, many bioscaffold-derived reports would become much more useful for QSAR/CADD if they were developed as larger congeneric matrices with standardized FXa assays and complete compound-level reporting. Their present limitation is often that the published evidence stops too early, before the scaffold has been tested as a true optimization platform.

Nevertheless, the current evidence base remains uneven. The isosteviol lineage provides the strongest natural-product-derived example, with exact FXa potency values, selectivity data, SPR or docking support, clotting readouts, pharmacokinetic information in selected cases, and ex vivo or in vivo antithrombotic evidence reported across representative studies [9,10]. Outside this lineage, however, available natural-product-derived FXa studies more often exhibit low or intermediate QSAR-readiness, because analog sets are smaller, translational readouts are sparser, and the resulting SAR is less suitable for local regression modeling than for qualitative scaffold-level interpretation [25,26].

From a practical standpoint, many natural-product-derived chemotypes should therefore be viewed primarily as sources of scaffold-level opportunity rather than as immediately modellable high-readiness QSAR datasets. Their strongest contribution lies in expanding the accessible design space for FXa inhibitor discovery and in identifying directions in which future semisynthetic programs may become more valuable through improved assay consistency, larger analog sets, and systematic translational characterization. The main natural-product-derived examples and their methodological value in this context are summarized in Table 5. This table, therefore, distinguishes structural inspiration from dataset reusability by pairing each representative scaffold with the most informative available potency or translational readouts.

## 6. Integrated QSAR-Readiness Map, Modellability, and Critical Gaps

### 6.1. Readiness as a Review-Level Triage Tool

The QSAR-readiness map is not intended to replace established QSAR validation rules or to rank chemotypes by intrinsic medicinal-chemistry value. Best practices already require curated structures, consistent endpoints, explicit definition of the applicability domain, robustness testing, and external validation whenever predictive models are claimed [28,29,30,31]. The present framework operates one step earlier, at the review level: it asks whether a published FXa inhibitor series contains enough chemically coherent, assay-comparable, and extractable information to justify local modeling after full curation. This triage function is important because nominally shared FXa activity alone does not define a modellable chemical dataset. At present, this framework has not been retroactively benchmarked against a uniform panel of published FXa QSAR models; formal back-testing would require re-curating primary datasets under common modeling and validation workflows and is therefore best treated as a next-step methodological study rather than as a claim made here. For the same reason, no weighted numerical score was imposed in this review: before common retrospective benchmarking becomes available, assigning fixed weights risks false precision and could understate the gating importance of endpoint quality, assay comparability, and activity-range adequacy.

Across the post-2014 literature examined here, QSAR-readiness is distributed asymmetrically but not in a simplistic way. Fully synthetic anthranilamide-centered and related series more often provide congeneric analog expansion, exact endpoints, and internally comparable assay formats. In contrast, isosteviol and other semisynthetic or natural-product-derived lineages contribute alternative topology and stereochemical diversity but more frequently require heavier curation or additional analog expansion [5,6,7,8,9,10,11,12,25,26,27,33,35,36,37,40]. This difference should be interpreted primarily as a consequence of the dataset’s architecture and reporting practices, not as evidence that bioscaffold-derived chemotypes are inherently less valuable. Table 6 summarizes how representative series were positioned within this review-level readiness map. In semi-quantitative terms, this pattern is directionally consistent with the broader evidence map of 15 post-2014 experimental reports and two retrospective or computational follow-up records, summarized in Section 2.1 [5,6,7,8,9,10,11,12,25,26,27,33,35,36,37,40,41]. In Table 6, it corresponds to one high-readiness lineage, one intermediate-to-high lineage, four intermediate-readiness series, two low-to-intermediate or borderline entries, and three low-readiness series. This distribution reflects multiple named scaffold families, including non-anthranilamide fully synthetic examples [5,6,11,27,37] and non-isosteviol natural-product-derived or semisynthetic examples [25,26,36], rather than a simple anthranilamide-versus-other-chemotypes comparison.

### 6.2. Why Some Series Are Modellable, and Others Are Not

#### 6.2.1. Scaffold Similarity, Endpoint Consistency, and Activity Range

One recurring pitfall in FXa literature analysis is treating compounds sharing a familiar binding motif as members of a single QSAR-eligible series. Apparent scaffold similarity can be superficial: anthranilamide-, benzamide-, or heterocycle-related inhibitors may differ substantially in linker geometry, stereochemical definition, conformational constraint, and spatial presentation of S1- and S4-directed substituents [2,4,13,23,24]. Pooling such compounds may increase nominal dataset size but decrease mechanistic coherence, because the model would combine related but non-equivalent binding solutions. For this reason, congenericity should be assessed not only by 2D scaffold labels but also by shared binding logic, endpoint consistency, and applicability domain boundaries [28,29,30,31,42].

For practical curation, our default position is conservative: compound sets should not be merged simply because they all inhibit FXa, or even because they all nominally address S1 and S4. Unless they share a sufficiently similar binding solution, endpoint definition, assay context, and activity range, the apparent gain in sample size may be bought at the cost of mechanistic noise. This is particularly problematic for ML workflows, where a larger but poorly harmonized dataset can look attractive while encoding assay artifacts rather than ligand–structure information. Mixing compounds with different substitution patterns or binding-pose assumptions can produce models that appear acceptable based on internal statistics but fail when tested outside the immediate training set. This is why local, mechanistically coherent datasets are preferable to larger but poorly harmonized FXa collections.

#### 6.2.2. Endpoint Heterogeneity and Curation Burden

Endpoint heterogeneity is an equally important barrier. Reported activity values may include K_i_, IC_50_, pK_i_, pIC_50_, single-point inhibition, threshold values, or censored readouts such as “>10 μm”. These formats remain useful for qualitative SAR, but they should not be merged into regression datasets unless endpoint type, assay format, censoring rules, and activity range are explicitly harmonized [28,29,30,31]. Secondary biological readouts, including selectivity panels, PT/aPTT, ex vivo effects, in vivo antithrombotic endpoints, or PK/ADME data, strengthen translational interpretation but should remain analytically distinct from primary FXa potency endpoints [5,6,9,10,12,33].

Activity-cliff-like behavior and geometry shifts provide a further reason to model locally rather than globally. Even within a congeneric series, small changes in S1 occupation, S4 aromatic-box engagement, or linker/core geometry may produce disproportionate changes in potency or selectivity [2,4,5,6,7,8,12,13,23,24,33]. Such discontinuities can be mechanistically informative, but they weaken the assumption that a single smooth descriptor–activity relationship can describe a broad or scaffold-drifting dataset. In practical FXa CADD, the safest rule is therefore to split early, model locally, explicitly define the applicability domain, and generalize only within the chemical and assay space represented by the curated series [28,29,30,31]. A practical example is provided by anthranilamide and cyclized anthranilamide-related lineages: although compounds such as **9b**, **16g**, and **8e** share recognizable FXa-directed design logic, their central-core and P1/P4 presentation are sufficiently different that they are better treated as related local subseries rather than as one automatically poolable regression set [7,8,12]. Compact high-potency sets, such as the pyrazolyl piperidines, illustrate the complementary problem: a potent lead can be highly informative medicinally yet still be too small for robust standalone QSAR modeling [37].

The main practical determinants of this local-modeling decision are summarized in Table 7. The table links each source of reduced readiness with its likely effect on QSAR interpretation and with a conservative analytical response so that the classification remains transparent rather than purely impressionistic.

Readiness determinants reflect the operational QSAR-readiness framework used in this review. They should be interpreted as pragmatic criteria for modellability rather than direct measures of medicinal-chemistry value or translational importance. Limited data-extraction confidence indicates that readiness placement is provisional and should not be treated as equivalent to classification based on full series-level extraction.

### 6.3. Critical Gaps and Future Directions

#### 6.3.1. From Potency Catalogs to Evidence-Aware Design

The main gap in the FXa inhibitor literature is not a lack of active compounds, but an uneven ability to convert reported activity into reusable design knowledge. Many series report promising potency yet provide limited information on activity distribution, assay comparability, breadth of selectivity, PK/ADME behavior, bleeding-relevant context, or complete structure-level curation [5,6,7,8,9,10,11,12,25,26,27,33,35,36,37,40]. A moderately novel scaffold with exact endpoints, sufficient analog density, and coherent assay conditions may therefore be more useful for CADD than a visually distinctive chemotype reported only as a small exploratory set. This is the practical meaning of QSAR-readiness in the present review. This helps explain why many potent academic leads remain translationally underdeveloped: potency is often demonstrated earlier and more completely than selectivity breadth, exposure, metabolic stability, interaction liability, bleeding-related pharmacology, or reproducible cross-series data structure. Many reported FXa inhibitors remain insufficiently characterized with respect to PK and ADME profiles. Without these data, it is difficult to assess whether a potent enzyme inhibitor has a realistic path to translation beyond early lead status.

#### 6.3.2. Reporting Standards and Balanced Scaffold Interpretation

A second gap concerns reporting standardization. Future FXa medicinal chemistry studies should report the enzyme source, substrate system, assay format, endpoint definition, concentration range, handling of censored data, full activity distributions, selectivity panel design, and unambiguous structures, including stereochemistry, salt form, and tautomeric assumptions, whenever relevant [28,29,30,31]. This point applies equally to synthetic and natural-product-derived programs. The comparatively cautious classification of several semisynthetic or bioscaffold-derived datasets reflects smaller analog sets, limited endpoint uniformity, or a higher curation burden in the published evidence, rather than an intrinsic limitation of natural-product chemistry itself [9,10,25,26,40]. Conversely, synthetic series should not be overvalued solely because they are chemically familiar; they require the same scrutiny of activity range, translational depth, and extraction confidence [5,6,7,8,11,12,33,35,37]. Detailed experimental information, including assay conditions, raw or compound-level activity values, and complete supplementary datasets, is still incomplete in many reports. This lack of reporting transparency remains one of the main practical barriers to reliable QSAR reuse.

#### 6.3.3. Benchmarking Against Marketed FXa Inhibitors

Benchmarking against approved direct FXa inhibitors is essential because marketed agents such as rivaroxaban, apixaban, and edoxaban set a translational standard that extends beyond enzymatic potency [2,3,4,14,15,16,17,18,19,20,21,22,32]. Early-stage experimental series should therefore be interpreted as lead-optimization or dataset-generating resources rather than as direct clinical alternatives unless they begin to address selectivity, exposure, metabolic liability, renal dependence, drug interaction risk, and bleeding-related pharmacology. This is particularly relevant for older adults with atrial fibrillation or venous thromboembolism, where frailty, multimorbidity, renal impairment, and polypharmacy shape the therapeutic window [14,16,17,18,19,20,21]. In this context, enzymatic FXa potency is a necessary but insufficient benchmark; predictable PK behavior and clinically interpretable safety margins are equally important design objectives. Table 8 is intended as a translational benchmark rather than a full clinical pharmacology review. Its main message for CADD-oriented discovery is that experimental FXa series must eventually be evaluated against the developability expectations already set by marketed oral anticoagulants and by non-marketed candidates that did not translate into routine clinical use. The table therefore separates clinical status, key pharmacological/developability considerations, and design-relevant lessons for early-stage FXa inhibitor discovery.

#### 6.3.4. Natural-Product-Derived Series: From Inspiration to Programmatic Expansion

Natural-product-derived FXa discovery should therefore shift from isolated scaffold inspiration to programmatic analog expansion. The isosteviol lineage demonstrates that a bioscaffold can support FXa-directed SAR, translational pharmacology, and computational reinterpretation when it is developed through a sufficiently coherent semisynthetic series [9,10,34,40,41]. Its value lies not only in potency but also in the potential to explore alternative topologies, stereochemical complexity, and multifunctional antithrombotic profiles. Future studies in this area would be strengthened by larger congeneric analog sets, standardized human FXa assays, direct selectivity panels, systematic PT/aPTT or anti-Xa readouts, early ADMET/PK evaluation, and prospective CADD-guided analog prioritization [9,10,25,26,34,40,41].

#### 6.3.5. AI/ML-Enabled CADD: Using Readiness as a Dataset Triage

The same readiness logic is also relevant to modern AI/ML workflows. Machine learning models can tolerate larger and more diverse datasets than classical local QSAR only when the underlying labels, structures, and assay contexts are curated with sufficient consistency. The framework can also support AI/ML-based FXa discovery by separating high-confidence regression-ready subsets from qualitative SAR material, flagging endpoint heterogeneity before model training, defining applicability-domain boundaries, and identifying which series are better suited for active learning, scaffold prioritization, docking rescoring, ADMET prediction, or generative design [28,29,30,31]. Nevertheless, because FXa-specific datasets remain limited and heterogeneous, AI/ML applications in this field are currently most useful for targeted tasks such as dataset triage, local modeling, docking rescoring, ADMET filtering, and prospective analog selection, rather than for broad global prediction models. Table 9 outlines how the same readiness triage can be used before applying contemporary AI/ML methods, including endpoint harmonization, training-set construction, active learning, docking rescoring, ADMET modeling, and generative design workflows, which are increasingly used in AI-driven drug discovery [43,44,45].

Thus, the framework can support contemporary ML workflows without overstating what the available FXa literature can deliver. Its role is to make dataset limitations visible before model training, not to replace experimental validation or formal model assessment. Under these conditions, AI/ML is most defensible as a support tool for focused prioritization, ADMET filtering, docking rescoring, or active-learning cycles, rather than as a broad global predictor of FXa potency. With small and heterogeneous datasets, global models are more likely to learn reporting and assay noise than to capture ligand–activity relationships robustly.

**Figure 2:**
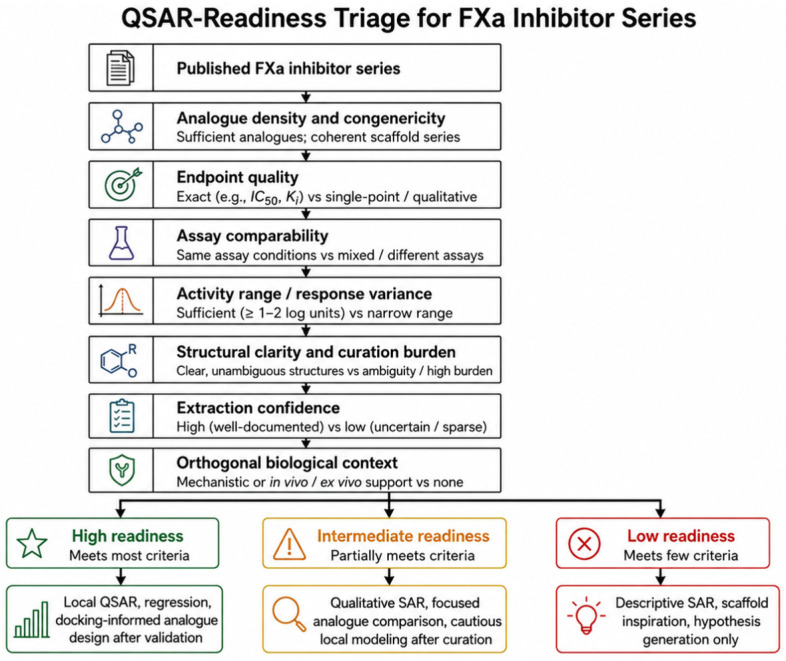
QSAR-readiness triage for published FXa inhibitor series.

Practically, future FXa CADD should proceed through four linked steps: (i) define chemically coherent subseries before descriptor generation; (ii) harmonize endpoints and assay context before modeling; (iii) keep enzymatic potency, selectivity, clotting, PK/ADME, and in vivo data as distinct but connected evidence layers; and (iv) use local models prospectively only within explicitly defined chemical and biological domains. Figure 2 summarizes this triage logic.

The workflow shown in Figure 2 evaluates series according to analog density and congenericity, endpoint quality, assay comparability, activity range or response variance, structural clarity and curation burden, extraction confidence, and orthogonal biological context. These criteria are used to assign a practical high-, intermediate-, or low-readiness category and to indicate the most appropriate analytical use, ranging from local QSAR or docking-informed analog design after validation to qualitative SAR, scaffold inspiration, or hypothesis generation. The scheme should be interpreted as a review-level triage tool, not as a substitute for formal QSAR model validation.

## 7. Conclusions

In this review, QSAR-readiness is used as a practical way to distinguish FXa inhibitor series that can support reproducible computational reuse from those better suited for qualitative SAR interpretation or scaffold inspiration. This distinction is important because dataset limitations should be made explicit before attempting QSAR, docking, AI/ML, or translational extrapolation. Small-molecule FXa inhibitor research remains scientifically productive, but its computational and translational value is highly uneven. Reported potency alone is not enough to make a series useful for downstream CADD. The most reusable datasets combine congeneric analog architecture, exact and internally comparable endpoints, sufficient activity range, clear structures, manageable curation burden, and at least partial translational context. Fully synthetic anthranilamide-centered and related scaffold families currently provide many of the more modellable examples, whereas natural-product-derived and semisynthetic series, especially isosteviol-derived chemotypes, contribute alternative topologies and scaffold diversity but often require additional analog expansion and reporting standardization before they can serve as high-confidence QSAR datasets. Thus, the most reusable FXa inhibitor datasets are not necessarily the most potent or visually novel ones, but those whose chemical and biological information can be reconstructed with confidence. We regard compact, heterogeneous, or incompletely reported series as valuable sources of scaffold inspiration, not as ready-made modeling inputs. Conversely, series that combine congeneric design, exact endpoints, and transparent assay context deserve priority for prospective CADD because they can support reproducible inference rather than retrospective storytelling.

The QSAR-readiness framework should therefore be viewed as a practical triage tool for dataset suitability, not as a substitute for formal model validation. It helps distinguish series suitable for local modeling from those better used for qualitative SAR, scaffold inspiration, or translational hypothesis generation. By aligning scaffold interpretation with assay quality, endpoint coherence, curation feasibility, and clinical benchmarking against established FXa inhibitors, future FXa discovery can move toward more reproducible, interpretable, and prospectively useful anticoagulant design.

## Figures and Tables

**Table 1:** Evidence-extraction domains and data-confidence criteria were applied throughout this review.

Domain	What Is Recorded	Why It Matters	Representative References
Series architecture	Core scaffold, series size, substitution pattern, analog progression, and degree of congenericity	Distinguishes well-defined medicinal-chemistry series from mixed or heterogeneous chemotype collections and supports the classification of local modellability	[5,7,8,12]
Primary FXa endpoint	K_i_, IC_50_, pK_i_/pIC_50_, percentage inhibition at a fixed concentration, threshold values, or censored values	Determines whether quantitative comparison across compounds is meaningful and whether activity data can support local SAR or QSAR-oriented analysis	[5,7,9,10,28,29,30,31]
Assay context	Enzyme source, substrate system, assay format, concentration range, and internal consistency of experimental conditions	Defines whether activity values can be compared within a series and whether cross-series pooling would be methodologically defensible	[5,9,10,12,28,29,30,31]
Activity range/response variance	Distribution and span of exact potency values within a series, proportion of censored values, and whether the series contains sufficient variation for informative ranking or regression	Determines whether a chemically coherent and assay-consistent dataset can support informative regression rather than only qualitative SAR interpretation	[28,29,30,31]
Selectivity profile	Activity versus thrombin and other related serine proteases	Informs target differentiation, mechanism, and bleeding-relevant pharmacological interpretation	[2,4,5,12,23,24]
Translational pharmacology	PT/aPTT, ex vivo effects, in vivo antithrombotic activity, bleeding observations, and antiplatelet or multi-endpoint pharmacology when reported	Links enzyme inhibition to broader anticoagulant behavior, but should be interpreted as complementary pharmacological evidence rather than as a direct substitute for primary FXa potency.	[5,6,9,10,12,33]
Developability signals	PK/ADME properties, solubility, permeability, metabolic stability, plasma exposure, bioavailability, or computational developability predictions	Supports scaffold prioritization beyond potency and helps connect medicinal-chemistry optimization with translational feasibility	[3,9,22,32,34]
Curation burden	Structural ambiguity, stereochemistry, salt form, tautomerism, compound identifiers, naming inconsistencies, and availability of complete series-level data	Affects descriptor reliability, reproducibility, and suitability for downstream computational modeling	[28,29,30,31]
Extraction confidence	Whether full series-level data were available, partially available, or limited to accessible publication metadata	Prevents overclassification of datasets when only abstract-level, publication-level, or incomplete series-level information was available	[28,29,30,31,35,36]

**Table 2:** Working framework for classifying QSAR-readiness at the series level.

Criterion	High Readiness	Intermediate Readiness	Low Readiness	Methodological/Illustrative References
Series size and congenericity	Approximately ≥20 closely related analogs within a single medicinal-chemistry core	Approximately 10–20 analogs or modest scaffold variation within one chemical family.	Fewer than 10 analogs or a heterogeneous set of weakly related scaffolds	[7,8,28,29,30,31]
Endpoint quality	Predominantly exact K_i_, IC_50_, pK_i_, or pIC_50_ values	Mixture of exact values and censored or semi-quantitative data	Mostly single-point inhibition data, threshold-only readouts, or sparsely reported activity values	[5,7,28,29,30,31,37]
Assay consistency	Same or clearly comparable FXa assay format across the series	Minor assay differences that remain partially comparable or potentially harmonizable.	Multiple non-equivalent assay settings that limit or prevent direct comparison	[28,29,30,31]
Activity range/response variance	Sufficient potency range to support informative regression and meaningful structure–activity discrimination	Moderate activity range that may support cautious local modeling or qualitative SAR.	Narrow or poorly distributed activity range that limits regression value despite apparent dataset cleanliness	[28,29,30,31]
Structural clarity and curation burden	Well-defined chemical structures with clear stereochemistry, salt form, and minimal ambiguity after routine curation	Some structural or annotation ambiguity, but largely resolvable without major information loss.	Significant ambiguity, undefined mixtures, unclear stereochemistry, or uncertain structure assignment	[28,29,30,31]
Data-extraction confidence	Full series-level data available for extraction, including structures, endpoints, assay context, and compound-level activity annotation	Partial but usable publication-level data available; some fields require cautious interpretation or verification.	Only limited metadata, abstract-level information, or incomplete series-level detail available; readiness placement should be regarded as provisional.	[28,29,30,31,35,36]
Orthogonal biological context	Selectivity, PT/aPTT, PK/ADME, ex vivo, or in vivo data available and interpretable as complementary evidence	Limited orthogonal biological or pharmacological characterization.	No information beyond isolated FXa inhibition data	[5,6,9,10,12,33]
Best analytical use	Local QSAR-oriented modeling, regression analysis, and docking-informed analog design after validation	Qualitative SAR analysis, focused analog comparison, and cautious local modeling after curation.	Descriptive SAR, scaffold inspiration, or hypothesis generation only	[28,29,30,31]

**Table 3:** Relationship between the present QSAR-readiness framework and established QSAR data-quality guidance. The table translates established QSAR curation and validation principles into the narrower review-level triage function used here [28,29,30,31].

Established QSAR/Data-Quality Principle	How It Is Translated in This Review	Practical Implications for FXa Inhibitor Literature
Chemical structure curation	Structures, stereochemistry, salt/tautomer state, and compound identifiers are checked for clarity and extraction confidence.	Ambiguous or only partially extractable series are retained for qualitative SAR but not upgraded to high readiness.
Endpoint and assay harmonization	K_i_, IC_50_, pK_i_/pIC_50_, single-point inhibition, and censored values are treated as non-equivalent unless the assay context is internally comparable.	Series with mixed endpoints or heterogeneous assay formats are not pooled into a single broad regression dataset.
Applicability-domain-aware modeling	Congenericity is interpreted as shared binding logic plus comparable chemistry, not only as a common scaffold label.	Local subseries are preferred over global FXa datasets when scaffold drift or different S1/S4 presentation is likely.
Robustness and external validation	Readiness is limited to suitability for future local modeling; it is not presented as proof of predictive performance.	A high-readiness placement means that modeling may be justified after curation and validation, not that a validated model already exists.
Data uncertainty and curation burden	Publication-level or metadata-level extraction is explicitly marked as provisional.	This reduces the risk of overinterpreting small, incompletely documented, or exploratory studies.

**Table 4:** Representative fully synthetic FXa inhibitor series and their practical value for translational SAR and QSAR-oriented analysis.

Chemotype/Series	Reference	What Makes It Useful	Translational Depth	Working Readiness
Isoxazolo[5,4-d]pyrimidin-4(5H)-ones	[5]	Focused synthetic series; compound **6g** showed IC_50_ = 0.013 μm, 2 × PT = 2.12 μm, and high selectivity over thrombin and trypsin	FXa inhibition, PT prolongation in human plasma, and selectivity over thrombin/trypsin	Intermediate
3,4-Diaminobenzoyl derivatives	[6]	Congeneric series with a clear FXa optimization strategy; compound **7b** was reported as a potent and selective direct FXa inhibitor	FXa inhibition, selectivity, and in vivo antithrombotic activity for lead compound **7b**	Intermediate
Anthranilamide derivatives	[7]	Fragment-based identification followed by analog expansion; compound **9b** showed an FXa IC_50_ of 23 nM	FXa inhibition, thrombin selectivity, and PT prolongation	Intermediate-to-high
Anthranilamide derivatives, expanded series	[8]	Expanded optimization series; compound **16g** combined potent/selective FXa inhibition with broader translational evaluation	In vitro FXa inhibition, in vivo antithrombotic activity, bleeding-risk evaluation, PK data, and cellular safety testing	High
Piperazinylanthranilamides	[35]	Anthranilamide-centered extension reported as a potential FXa inhibitor series; useful for qualitative SAR, but quantitative reuse should remain conservative because only accessible publication-level information could be extracted in this review	FXa inhibition, thrombin selectivity, and rat anticoagulant activity are reported in accessible publication metadata.	Low-to-intermediate
Pyrazolyl piperidines	[37]	Compact pyrazolyl piperidine series; compound **4a** showed IC_50_ = 13.4 nM against FXa	FXa inhibition with PT/aPTT clotting readouts for selected compounds	Low
Anthranilamide-based FXa inhibitors	[33]	Selective anthranilamide-based series with exact inhibitory values and thrombin selectivity	FXa inhibition, thrombin selectivity, and PT prolongation in plasma	Intermediate
2,3-Dihydroquinazolin-4(1H)-ones	[12]	Cyclized anthranilamide-related scaffold; compound **8e** showed IC_50_ = 21 nM against FXa and thrombin IC_50_ = 67 μm	FXa inhibition, thrombin selectivity, PT/aPTT, AV-shunt activity, and bleeding-risk evaluation	Intermediate-to-high
Tetrahydropyrazolopyridones	[11]	Compact synthetic series; compound **15c** showed IC_50_(FXa) = 0.14 μm and strong enzymatic FXa inhibition	FXa inhibition, anticoagulant activity, and selected in vivo venous thrombosis evaluation	Low-to-intermediate
Dual FXa/XIa pyrroloquinolinone hybrids	[27]	Dual-target exploratory design probing FXa/XIa activity relationships with thrombin counter-screening	FXa/XIa screening with thrombin counter-screening	Low

**Table 5:** Representative natural-product-derived and semisynthetic FXa-related chemotypes and their practical value for translational SAR and QSAR-oriented analysis.

Chemotype/Series	Reference	What Makes It Useful	Translational Depth	Working Readiness
Isosteviol-based antithrombotic lead series	[9]	Semisynthetic diterpenoid scaffold; compound **6k** selectively inhibited FXa with K_i_ = 0.015 μm	FXa inhibition, selectivity against a serine-protease panel, docking, ex vivo PT/aPTT prolongation, antiplatelet activity, PK data, and ex vivo/in vivo antithrombotic activity	Intermediate-to-high
Isosteviol derivatives evaluated as FXa inhibitors	[10]	Focused semisynthetic analog series; compounds **22**, **35**, and **38** showed stronger FXa inhibition than isosteviol	FXa inhibition, SPR evaluation for selected compounds, and PT/aPTT activity for compounds **22** and **35**	Intermediate
Isosteviol dataset, QSAR reinterpretation	[40]	Demonstrates that natural-product-derived series can support QSAR-oriented analysis when structurally congeneric	Retrospective QSAR analysis of experimentally reported isosteviol-related FXa inhibition data	Intermediate
ent-Norstrobane diterpenoids	[25]	Diterpenoid scaffold rearrangement; compound **7** showed IC_50_ = 81 ± 11 nM against FXa	Enzymatic FXa inhibition with limited additional translational data	Low-to-intermediate
Epoxy-pimarane diterpenoids from kirenol	[26]	Expands diterpenoid design space beyond isosteviol; reported FXa IC_50_ values ranged from 0.22 to 27.9 μm	Enzymatic FXa screening with moderate activity; edoxaban used as positive control	Low
4,7-Dihydroxycoumarin derivatives	[36]	Natural-product-related scaffold evaluated across multiple coagulation factors; quantitative interpretation should be approached with caution, given the limited set of available analogs.	Multi-target coagulation-factor testing with limited analog-set depth based on available information	Low

**Table 6:** Conceptual placement of representative FXa inhibitor chemotypes within the QSAR-readiness framework and their recommended analytical use.

Series/Chemotype	Domain	Readiness Tier	Main Reason for Placement	Best Analytical Use
Anthranilamide derivatives [7]	Fully synthetic	Intermediate-to-high	Interpretable common core, fragment-based design logic, exact FXa potency, thrombin selectivity, and PT readout for the lead compound	Local SAR, scaffold-focused follow-up design, and constrained QSAR-oriented analysis
Anthranilamide derivatives, expanded series [8]	Fully synthetic	High	Larger congeneric optimization series with exact inhibitory data, clear FXa-focused scaffold logic, in vivo antithrombotic activity, bleeding-risk evaluation, and PK data	Local QSAR, descriptor-based modeling, and docking-informed analog prioritization
Anthranilamide-based FXa inhibitors [33]	Fully synthetic	Intermediate	Selective anthranilamide-based series with exact inhibitory values, thrombin selectivity, and in vitro anticoagulant activity for selected compounds	Qualitative SAR, local scaffold-focused comparison, and support for anthranilamide design logic
3,4-Diaminobenzoyl derivatives [6]	Fully synthetic	Intermediate	Congeneric series with a clear FXa optimization strategy; compound **7b** was reported as a potent, selective direct FXa inhibitor with in vivo antithrombotic activity	Qualitative SAR, focused analog comparison, and translational SAR interpretation.
Piperazinylanthranilamides [35]	Fully synthetic	Low-to-intermediate	Anthranilamide-centered extension with reported FXa inhibition and thrombin selectivity; placement is provisional because detailed series-level extraction was limited to accessible publication-level information	Qualitative SAR and cautious hypothesis-generating comparison; not definitive local QSAR classification
Isoxazolo[5,4-d]pyrimidin-4(5H)-ones [5]	Fully synthetic	Intermediate	Focused synthetic series; compound **6g** showed IC_50_ = 0.013 μm, 2 × PT = 2.12 μm, and high selectivity over thrombin and trypsin	Series-level SAR, docking-informed interpretation, and constrained local modeling
Isosteviol derivatives [9,10,40]	Natural-product-derived/semisynthetic	Intermediate	Strong semisynthetic isosteviol lineage with exact FXa potency, translational readouts, and retrospective QSAR reinterpretation, but higher curation burden than simpler synthetic series	Semisynthetic SAR, scaffold-specific CADD after curation, and focused QSAR reinterpretation.
Pyrazolyl piperidines [37]	Fully synthetic	Low	Compound **4a** showed an IC_50_ of 13.4 nM and PT/aPTT readouts, but the analog count remains too small for robust regression modeling.	Qualitative SAR and lead-inspiration analysis
4,7-Dihydroxycoumarin derivatives [36]	Natural-product-related	Low	Available information indicates a very limited set of derivatives with multi-target coagulation readouts, insufficient for local QSAR.	Mechanistic hypothesis generation and scaffold inspiration
Tetrahydropyrazolopyridones [11]	Fully synthetic	Low-to-intermediate/intermediate	Congeneric synthetic series with reported FXa inhibition and selected in vivo venous thrombosis evaluation; modellability depends on endpoint coverage and extractable activity range	Qualitative SAR, constrained local modeling, and translational SAR interpretation.
Dual FXa/XIa pyrroloquinolinone hybrids [27]	Fully synthetic/dual-target exploratory	Low	Dual-target exploratory design with FXa/XIa screening and thrombin counter-screening; not suitable for a clean single-target FXa QSAR model	Multi-target hypothesis generation and qualitative SAR

**Table 7:** Determinants of QSAR-readiness in published FXa inhibitor datasets and recommended analytical responses.

Readiness Determinant	What It Looks Like in Practice	Effect on QSAR-Readiness	Best Analytical Response	Supporting References
High congenericity with exact endpoints	One medicinal-chemistry core, clear analog progression, predominantly exact K_i_/IC_50_ values, and internally consistent assay conditions	Supports reliable local QSAR modeling, interpretable SAR trends, and docking-informed prioritization	Retain as a single local series; validate carefully and preserve assay consistency.	[7,8,28,29,30,31]
Moderate scaffold drift within one family	Shared design intent but noticeable changes in linker geometry, ring system, or substituent vectors across subsets	Reduces the coherence of a global model while still allowing for qualitative SAR interpretation	Split into subseries or apply cautiously defined local QSAR models.	[11,12,28,29,30,31]
Endpoint heterogeneity	Mixture of K_i_, IC_50_, pK_i_/pIC_50_, single-point inhibition, and censored values within one dataset	Introduces artificial variance and reduces the interpretability of regression-based QSAR models	Restrict analysis to one endpoint class or treat the remaining data qualitatively.	[7,28,29,30,31,37]
Activity-cliff-like behavior and geometry shifts	Minor structural changes produce disproportionate differences in potency, selectivity, or translational readouts.	Disrupts the continuity required for smooth QSAR relationships, even within congeneric series	Use matched-pair analysis, local modeling, and discontinuity-focused SAR interpretation.	[2,4,13,23,24,28,29,30,31]
High curation burden	Ambiguous stereochemistry, unresolved salt forms or tautomers, inconsistent compound identifiers, and discrepancies between reported structures	Reduces the descriptor reliability and reproducibility of computational models	Perform rigorous curation or exclude from quantitative modeling workflows.	[28,29,30,31]
Limited data-extraction confidence	Full series-level structures, assay details, or compound-level activity annotations are unavailable or accessible only through limited publication metadata.	Makes readiness placement provisional and increases the risk of overclassifying a dataset as modellable	Classify conservatively; use for qualitative SAR unless full series-level extraction can be verified.	[28,29,30,31,35,36]
Rich translational pharmacology with limited series coherence	PT/aPTT, in vivo, or PK data reported for small, semisynthetic, or structurally complex analog sets	Enhances biological interpretation but does not directly improve QSAR suitability	Use for translational SAR and medicinal-chemistry decision-making, not as a pooled QSAR dataset.	[9,10,12,28,29,30,31]

**Table 8:** Clinical status and benchmark context for direct FXa inhibitors relevant to early-stage FXa medicinal chemistry.

Agent/Class	Clinical Status and Main Use Context	Key Pharmacological/Developability Considerations	Design-Relevant Benchmark for New FXa Inhibitor Series
Rivaroxaban	Approved/marketed oral direct FXa inhibitor; used across major thromboembolic indications, including stroke prevention in atrial fibrillation and VTE treatment or prevention [2,3,14,17,18,19,20,21,22,32].	Oral direct FXa inhibition with clinically established anticoagulant use; interpretation of new series should consider renal function, interaction liability, exposure predictability, and bleeding-risk context.	Sets a benchmark for oral FXa inhibition, predictable anticoagulation, and clinically manageable dosing, while still requiring attention to renal function, drug interactions, and bleeding risk in older or multimorbid patients.
Apixaban	Approved/marketed oral direct FXa inhibitor with broad use in atrial fibrillation and VTE-related indications [3,4,14,17,18,19,20,21,22,32].	Oral FXa inhibition with a clinically established balance of potency, exposure, and safety; population-specific factors such as renal function, concomitant therapy, and bleeding vulnerability remain important comparators.	Provides a clinically established reference for balancing potency, selectivity, oral exposure, and safety; early series should not be judged by enzyme potency alone.
Edoxaban	Approved/marketed oral direct FXa inhibitor in many regions; clinically used for atrial fibrillation and VTE treatment contexts [3,14,17,18,19,20,21,22,32].	Oral FXa inhibition in AF/VTE-related settings: renal function dependence, systemic exposure, and interaction risk are key developability considerations when comparing with experimental compounds.	Highlights the importance of exposure predictability, renal-function considerations, and clinically interpretable efficacy–safety trade-offs.
Betrixaban	Approved in the United States for extended VTE prophylaxis in acutely ill medical patients, with regional availability and regulatory status differing from the more widely used oral FXa inhibitors [22,32].	Indication-specific clinical use illustrates that the exposure profile, renal elimination considerations, and regulatory context can strongly shape the practical value of an FXa inhibitor.	Illustrates that indication selection, renal elimination profile, half-life, and regulatory context can be as important as biochemical target engagement.
Representative non-marketed clinical candidates	Earlier clinical FXa inhibitor candidates such as darexaban, otamixaban, letaxaban, or eribaxaban progressed beyond discovery but did not become routinely marketed anticoagulants [22,32].	These candidates illustrate that biochemical FXa inhibition and clinical progression do not guarantee translation when the route, exposure, bleeding liability, efficacy-safety balance, or population-specific utility is insufficiently favorable.	Their trajectories emphasize that FXa potency and even clinical progression are insufficient without a favorable efficacy, bleeding, exposure, route-of-administration, and population-specific risk–benefit profile.

**Table 9:** Practical uses of QSAR-readiness triage in AI/ML-enabled FXa inhibitor discovery. The table synthesizes general QSAR data-curation principles and selected AI/ML drug-discovery applications with FXa-focused dataset triage [28,29,30,31,43,44,45].

AI/ML Task	Dataset Requirement	How Readiness Triage Helps
Regression modeling of potency	Exact, harmonized K_i_/IC_50_ or transformed pK_i_/pIC_50_ values with sufficient response variance.	Separates regression-ready local subsets from qualitative or censored activity data.
Classification or prioritization	Clear active/inactive thresholds and assay context that justify label assignment.	Prevents arbitrary binning of compounds measured under incompatible assay formats.
Docking rescoring/consensus CADD	Chemically coherent series with plausible shared binding mode and interpretable S1/S4 engagement.	Reduces the risk that docking scores are trained or interpreted across non-equivalent binding solutions.
Active learning and analog selection	Constrained chemical domain with reliable labels and feasible prospective analogs.	Identifies series in which new compounds would genuinely reduce model uncertainty rather than introduce noise.
Generative or scaffold-hopping workflows	Explicit boundaries between modellable local SAR and exploratory scaffold inspiration.	Keeps generative suggestions tied to the applicability domain and flags when experimental validation is essential.
ADMET and translational filtering	Potency data linked to selectivity, PT/aPTT, PK/ADME, or bleeding-relevant signals where available.	Encourages multilayer decision-making without merging mechanistically distinct endpoints into one undifferentiated model.

## Data Availability

No new data were created or analyzed in this study. Data sharing is not applicable to this article.
